# Severe Iron Deficiency Anemia Leading to Thrombocytosis With Arterial and Venous Thrombosis

**DOI:** 10.7759/cureus.17893

**Published:** 2021-09-11

**Authors:** Deepak Venugopalan Pathiyil, Renoy A Henry, Jimmy Joseph, Akash T Oomen, Jyothi Janardhanan Kakkra

**Affiliations:** 1 Internal Medicine, Amrita School of Medicine, Kochi, IND

**Keywords:** thrombosis, secondary/reactive thrombocytosis, crohn’s disease (cd), gastrointestinal infection, splenic infarcts

## Abstract

A 45-year-old female who was a teacher by profession with a history of chronic asymptomatic anemia in the past presented to our hospital with complaints of intermittent fever for two weeks, cough with expectoration, dyspnea on exertion, and left upper limb edema for four days. She had a history of abdominal pain after food intake. She gave a history of having anemia for the past 23 years. Evaluation after admission showed raised inflammatory markers, marked thrombocytosis, and severe iron deficiency anemia. Further imaging in the form of a CT of the abdomen and thorax showed that she had a left-sided pleural effusion which showed an exudative picture, splenomegaly with a splenic infarct with a splenic abscess, and a suprarenal abdominal aorta thrombus. She was also found to have deep vein thrombosis (DVT) of the left subclavian and proximal internal jugular vein in a ultrasonogram (USG) Doppler. The workup done ruled out congenital and acquired causes of thrombosis and after extensive evaluation the patient was found to have unexplained thrombosis. The cause of unexplained thrombosis is the point of interest in this patient. Despite extensive workup, no precise cause for thrombosis, which was both arterial and venous in nature could be found out initially. Hence by exclusion, the possibility of secondary thrombocytosis causing the thrombosis was considered. Over the next few years, this patient underwent repeat esophageal endoscopies, colonoscopies, and capsule studies all without being able to pinpoint a diagnosis. Eventually three years later, a CT enteroscopy with biopsy showed the diagnosis of Crohn’s disease and the patient was started on appropriate immunosuppressive treatment for the same. There have been multiple case reports of thrombocytosis causing arterial or venous thrombus but not many have recorded both arterial and venous thrombosis in the same patient.

## Introduction

Anemia, especially iron deficiency anemia is an extremely common finding in this world. According to a study published recently in The Lancet by Pasricha et al. [1], it has been understood that anemia affects nearly 33% of the world’s non-pregnant women and about half the cases are due to iron deficiency. Women of childbearing age are particularly affected and it unfortunately leads to not just physical but significant emotional and psychological issues as well. Unfortunately, iron deficiency anemia remains an issue which is largely ignored or treated with simple iron replacement without really looking into the larger issue of the cause for the anemia.

In most situations it can be easy to pinpoint the cause of anemia but there are instances where this may be challenging. This case report focuses on secondary thrombocytosis caused due to severe iron deficiency anemia. While thrombocytosis secondary to iron deficiency anemia has been considered a common and largely benign condition, it has an association with stroke [2] and retinal vein occlusions [3]. The concept of a large number of platelets naturally causing a prothrombotic state is being understood more with newer evidence coming into light [4].

The final point of focus is the need for sustained efforts in identifying an underlying diagnosis for patients with iron deficiency anemia. While the treatment and immediate recovery with iron replacement might be straightforward, it is always important to rule out any sinister cause which can rear its ugly head in the future. In summary, iron deficiency anemia is an issue that plagues a large part of the world’s population, has the potential to cause significant complications, and needs detailed efforts in order to identify an underlying cause.

## Case presentation

The patient was a 45-year-ld female and a teacher by profession. She had no documented comorbidities except for a history of chronic anemia since the age of 21. As she remained asymptomatic from her anemia, she did not seek evaluation. She presented with intermittent fever and worsening dyspnea on exertion for the last three weeks and left upper limb edema since four days. Prior to presenting in our hospital, she was admitted and worked up in another hospital. There was significiant anemia and thromobocytosis on her complete blood count. The liver, renal, and thyroid function tests were normal and the peripheral blood smear showed microcytic hypochromic anemia with thrombocytosis. An ultrasound scan of the abdomen showed a mild fatty liver with minimal splenomegaly with multiple irregular hypoechoic lesions in the spleen. There was also a dilated small bowel and enlarged cystic right ovary. A CT abdomen showed splenomegaly with diffuse nonenhancing hypodensities and scarce normal enhancing parenchyma suspicious of extensive infarct which could be acute/subacute. There was also a suspicion of focal irregular filling defect in upper abdominal aorta suggestive of a thrombus. There was also a right ovarian cyst and left-sided moderate pleural effusion with basal consolidation. Her bone marrow aspiration and biopsy showed a normal picture.

On examination, the patient was conscious, oriented and hemodynamically stable. She had significant pallor, bilateral pitting edema on both lower limbs and she also left upper limb edema. The left upper limb was tender and red. There was no icterus or cyanosis. Her tongue was fissured and dry. Abdominal examination revealed a mildly enlarged tender spleen. Bowel sounds were present. Her respiratory examination showed that air entry was absent on the left side with a stony dull percussion note. Her cardiovascular and nervous system were within normal limits. A vast array of blood tests were done at admission and during initial workup and are detailed in Table 1. 

**Table 1:** Laboratory investigations. MCV, mean corpuscular volume; LDH, lactate dehydrogenase; ANA, antinuclear antibody

Laboratory investigation	Patient value	Normal value
Hemoglobin	6.5 (g/dL)	11.5-16.5 (g/dL)
White blood count	13,900 (cells/mm3)	4,000-11,000 cells/mm3
Platelets	8.8 (lakhs/μL)	1.3-4.2 (lakhs/μL)
MCV	59.1 (fL)	72-96 (fL)
Red cell distribution width	26.7%	11.5%-15%
C reactive protein	134.7 (mg/L)	0-5 (mg/L)
Erythrocyte sedimentation rate	59 (mm/h)	0-20 (mm/h)
Liver function tests	Albumin/globulin reversal	
Renal function tests	Normal	
Serum iron level	6.9 (mcg/dL)	60-180 (mcg/dL)
Total iron binding capacity	147.7 (μg/dL)	250-450 (μg/dL)
Ferritin	80 (ng/mL)	12-150 (ng/mL)
Transferrin saturation	4.69%.	20%-50%
Serum LDH level	696.2 (IU/L)	140-280 (IU/L)
Direct Coombs test	1+ Positive	
Indirect Coombs test	Negative	
C4	30 (mg/dL)	12-42 (mg/dL)
C3	124 (mg/dL)	80-178 (mg/dL)
Anti ds-DNA	Negative	
ANA profile	Negative	
Beta 2 glycoprotein	Negative	
Lupus anticoagulant	Negative	
Anti cardiolipin	Negative	

Bi-directional endoscopy revealed antral gastritis and external hemorrhoids. Biopsies taken at this point were normal. Hence, the impression of her anemia was likely secondary to iron deficiency.

**Figure 1:**
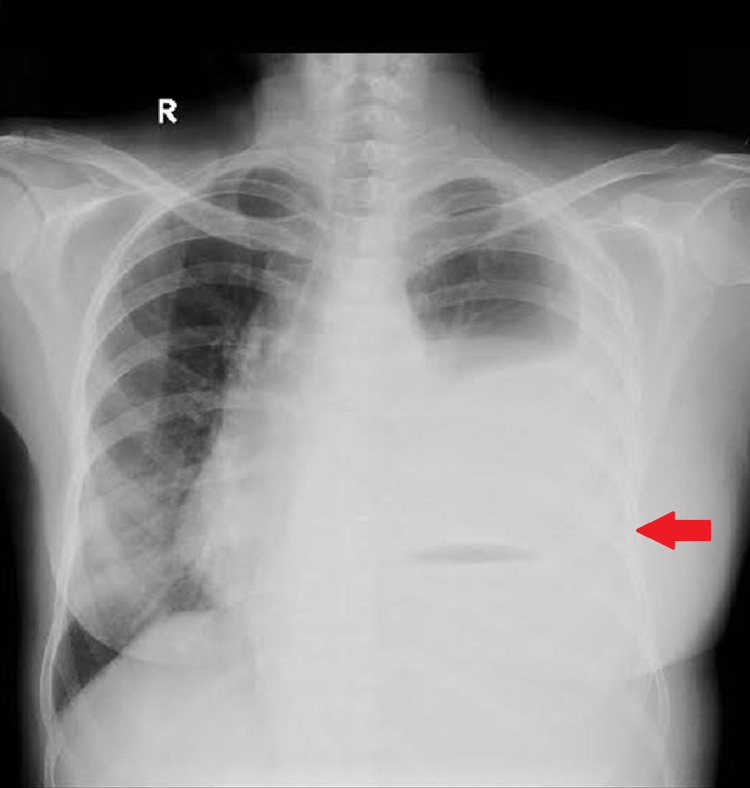
Chest X-ray showing a left-sided pleural effusion.

**Figure 2:**
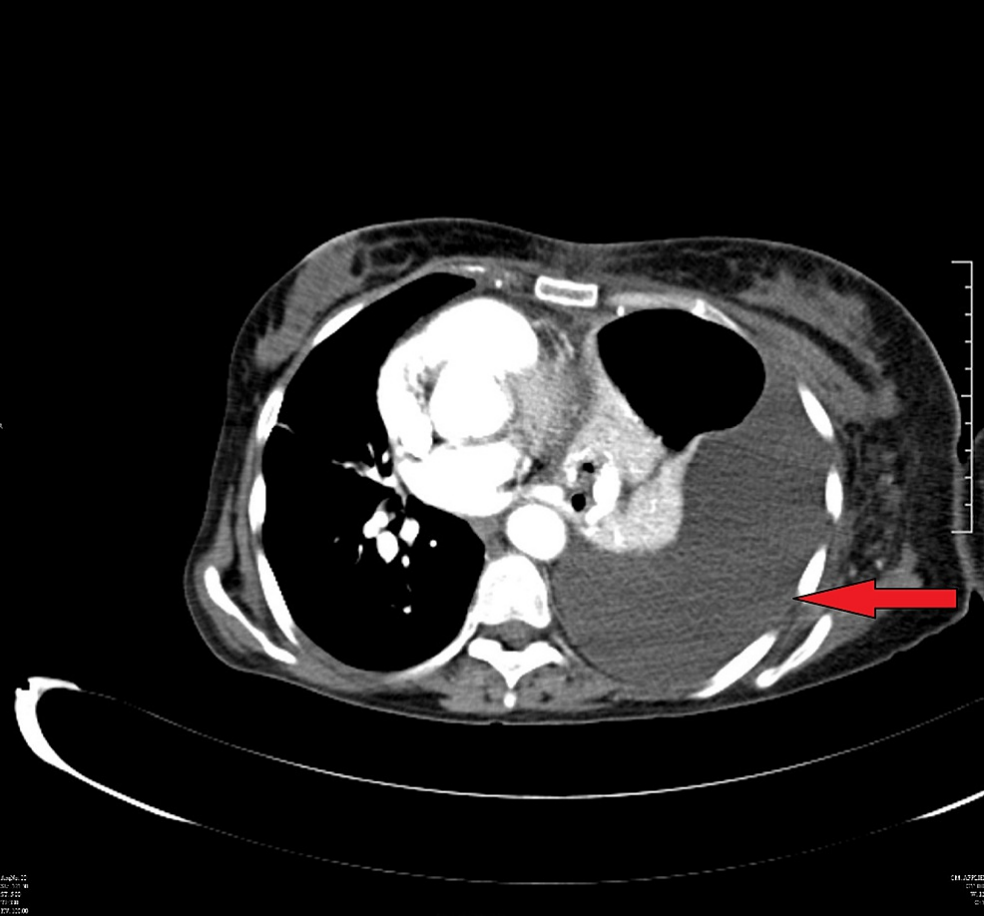
CT chest showing the left-sided pleural effusion.

**Figure 3:**
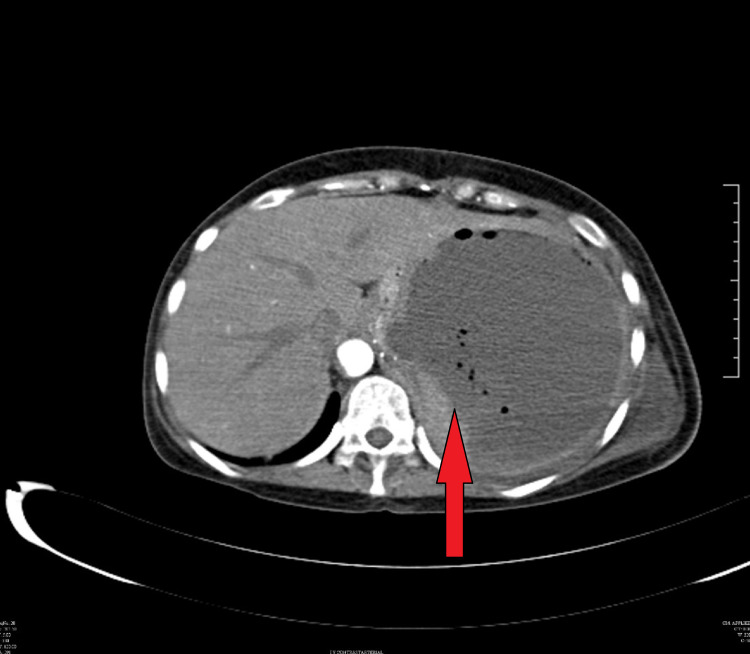
CT abdomen showing splenic abscess with air foci.

**Figure 4:**
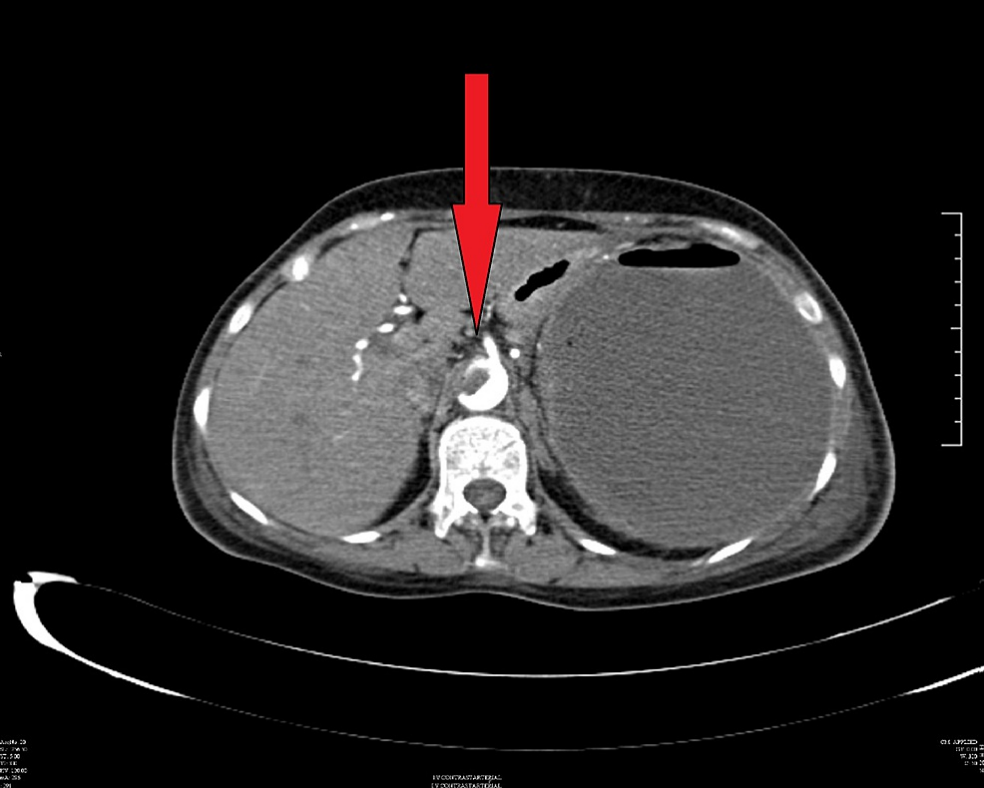
CT abdomen showing a filling defect in the abdominal aorta which is suggestive of a thrombus.

Her chest X-ray showed the presence of a left sided pleural effusion (Figure 1) and a CT chest and abdomen with contrast was done which showed a left sided pleural effusion (Figure 2) and the entire spleen was replaced by a necrotic collection measuring 13 cm x 11 cm (Figure 3). This collection showed air foci within. Abdominal aorta showed an eccentric filing defect involving the suprarenal aorta (Figure 4). A splenic artery filling defect was also seen. This was reported as findings consistent with an abdominal aortic thrombus, a splenic artery infarct, and a splenic abscess probably secondary to the infarct. 

 

 

As she was having significant edema of the left upper limb, a Doppler of her upper limbs was also done. It showed that the radial, ulnar, brachial, axillary vein, subclavian and proximal part of the internal jugular vein showed no flow augmentation or compressibility and showed echogenic thrombus within. These were consistent with the findings of a DVT of the left upper limb veins. The pleural fluid analysis of the left sided effusion showed total count of 300 (cells/mm3) (N60L40), BF-Protein 3.01, BF-LDH 333.3 (IU/L), BF-Glucose 102.6, and BF-ADA 7.2 (less than 40 U/L). This was conclusively an exudative picture based on lights criteria. Various tests were done on the pleural fluid including a Gram smear, acid-fast bacillus (AFB) smear, fungal smear, AFB culture, GeneXpert, routine culture and it all came back as negative. The cytology analysis was also negative.

A CT guided pigtail was put by the gastroenterology team in order to drain the splenic abscess. Some 1.5 L of frank pus was drained and she was started on broad spectrum antibiotics. The pus that drained via the drain was sent for Gram smear, AFB smear, fungal smear, AFB culture, GeneXpert, routine culture and it all came back as negative. She was also started on low molecular weight heparin (LMWH) and was bridged with warfarin to combat the arterial and venous thrombi. She was started on oral iron supplements. Malignancy markers (Ca 125, Ca 15-3, Ca 19-9, carcinoembryonic antigen, CEA) were checked and were all normal except for a mildly raised Ca 125 level of 114.5 U/mL (less than 46 U/mL). A pap smear and trans vaginal ultrasound were done which were both normal. After discussion with Hematology and Gynecology, this was opined to be a likely false positive level due to the splenic abscess. A V617F JAK2 mutation analysis was also done and was negative. Congenital causes for thrombocytosis were also considered such as Factor V Leiden mutation, protein C&S deficiency, and hyperhomocysteinemia and were all normal. A capsule endoscopy showed no evidence of small bowel disease.

**Figure 5:**
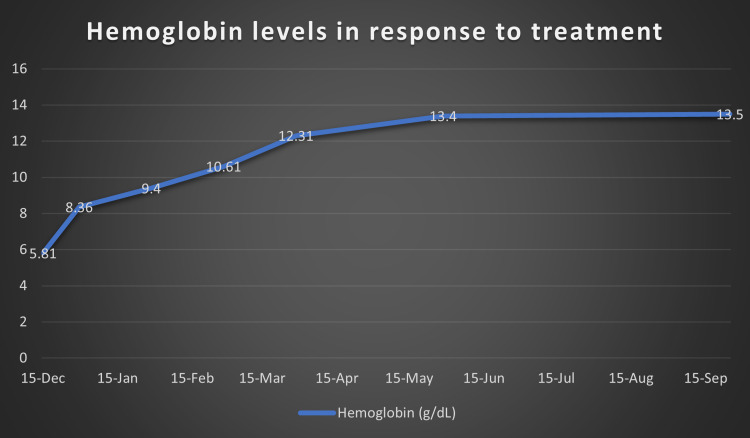
Hemoglobin levels (g/dL) over nine months after initiating on iron supplements.

**Figure 6:**
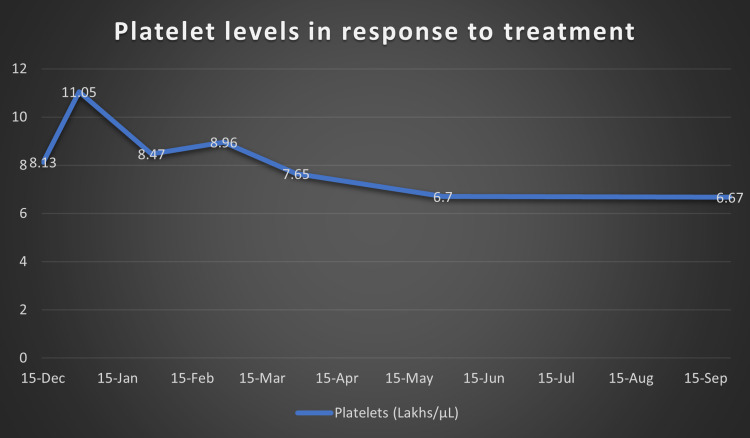
Platelet levels (lakhs/μL) over nine months after initiating treatment of iron deficiency anemia.

**Figure 7:**
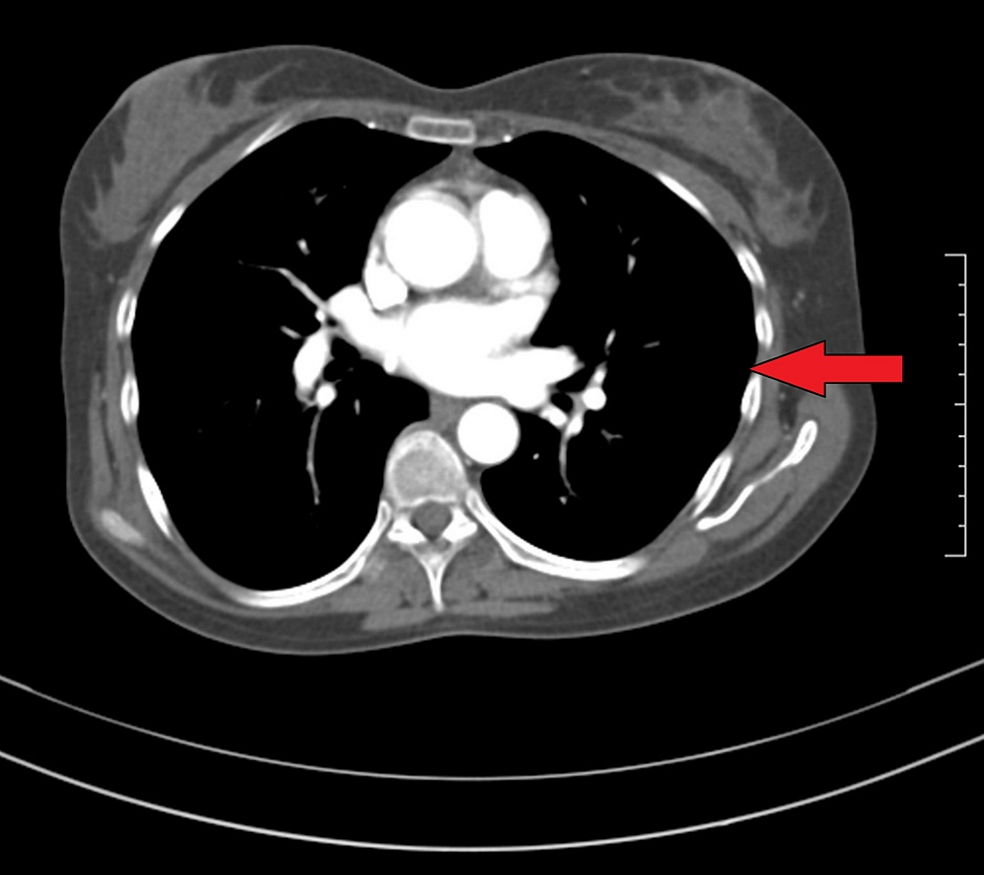
CT chest showing resolved left-sided pleural effusion after six months.

**Figure 8:**
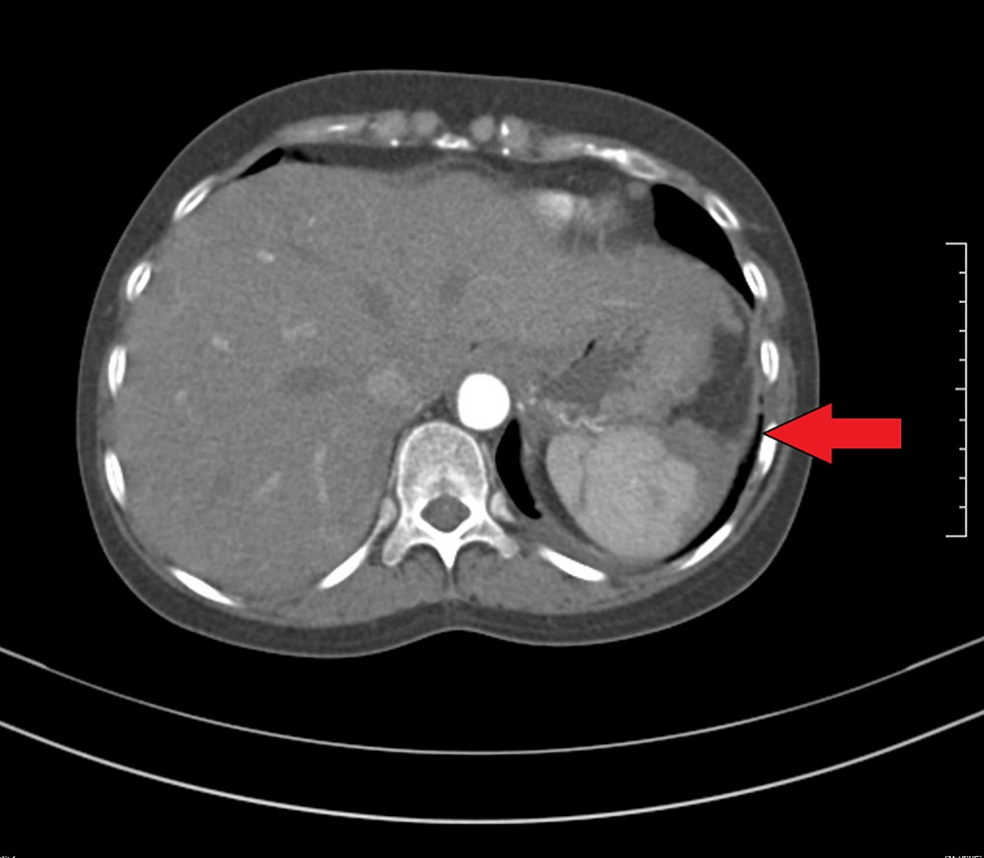
CT abdomen showing significantly resolved splenic abscess six months after treatment.

At this point, after a multi-disciplinary team meeting involving Hematology, Gastroenterology, Infectious disease and Internal Medicine, it was decided that the cause of her symptoms was secondary to iron deficiency anemia. As no cause for anemia was identified, we attributed it to possible long-standing menorrhagia and dietary deficiency. We then attributed the iron deficiency anemia to be causing reactive thrombocytosis. As procoagulant workup was also negative, the thrombocytosis was considered to be the cause for her arterial and venous thrombosis. Hence, we started treating her iron deficiency anemia with initially IV and subsequently oral replacement. We also continued anticoagulation for the thrombosis and continued antibiotics for a period of two weeks. Over the next few months, she made significant progress both clinically and in terms of her lab reports. Figure 5 shows improvement in the patient’s hemoglobin levels post treatment with iron supplements and Figure 6 shows the normalizing of platelet levels corresponding to the improving hemoglobin levels during this time. Figures 7-8 also shows a normal CT chest and abdomen picture six months after initiation of treatment, with complete resolution of pleural effusion and splenic abscess. 

Over the next three years the patient led a relatively normal life which was intermittently punctuated by worsening of anemia. Hence, the patient had yearly endoscopies, colonoscopies, and CT and capsule studies to ascertain any possible cause for her anemia. All of them were normal until the patient eventually underwent a push enteroscopy and biopsy. The patient was initially apprehensive about the risks of the procedure but after finally undergoing the intervention, the evidence of Crohn's disease was revealed. She was hence started on immunosuppressive medication and currently continues to be on the same. She has not had any further adverse issues.

## Discussion

The cause of unexplained thrombosis is the point of interest in this patient. According to Harrison's Principles of Internal Medicine (2nd edition) [5] the most likely causes of a patient having both arterial and venous thrombosis are as below. The reason why any particular differential diagnosis was ruled out has also been mentioned alongside (Table 2).

**Table 2:** Causes for arterial and venous thrombosis with reason for exclusion. APLA, antiphospholipid antibodies

Causes for arterial and venous thrombosis	Reason for exclusion
APLA	The patient’s APLA tests including beta 2 glycoprotein, and cardiolipin were negative
Hyperhomocysteinemia	Her homocysteine levels were normal
Paroxysmal nocturnal hemoglobinuria	Bone marrow studies were not suggestive and CD55, CD59 were normal
Malignancy	Radiology, cytology, and tumor marker screen negative
Bechet’s disease	She had no mouth or genital ulcers and autoimmune workup was negative

Hence, as direct causes for the arterial and venous thrombosis were ruled out, we proceeded to focus on any cause for general thrombophilia. Again, based on the causes for thrombophilia we divided it into congenital and acquired. The causes of why each differential was ruled out is again mentioned alongside (Tables 3-4).

**Table 3:** Workup regarding congenital causes for thrombophilia.

Congenital causes for thrombophilia	Status in patient
Factor V deficiency (Leiden)	Negative
Prothrombin mutation	Negative
Antithrombin III deficiency-	Negative
Protein S and Protein C deficiency	Negative

**Table 4:** Workup regarding acquired causes of thrombophilia. GI, gastrointestinal

Acquired causes for thrombophilia	Status in patient
Antiphospholipid syndrome	Negative
Paroxysmal nocturnal hemoglobinuria	Negative in bone marrow and CD 55, 59 normal
Sickle cell disease	Negative
Polycythemia vera	Negative
Essential thrombocytosis	Possible
Reactive thrombocytosis	Possible
Malignancies	Solid organ, GI or blood. (Particularly metastatic) - all imaging and biopsies negative

Having ruled out congenital and acquired causes, the essential thrombocytosis criteria were considered. This required the presence of both A criteria together with B3 to B6, or of criterion A1 together with B1 to B6 (Table 5).

**Table 5:** Criteria for essential thrombocytosis.

A1. Platelet count > 450 × 103/µL for at least two months
A2. Acquired V617F JAK2 mutation present
B1. No cause for a reactive thrombocytosis
B2. No evidence of iron deficiency
B3. No evidence of polycythaemia vera
B4. No evidence of chronic myeloid leukemia
B5. No evidence of myelofibrosis
B6. No evidence of a myelodysplastic syndrome

Clearly, this patient did not satisfy the criteria for essential thrombocytosis. Hence, the possibility of iron deficiency anemia causing secondary thrombocytosis leading to thrombosis was considered the main diagnosis. There have been multiple case reports of thrombocytosis causing arterial or venous thrombus but only isolated reports have recorded both arterial and venous thrombosis in the same patient. The cause of thrombosis in patients with thrombocytosis has also not been conclusively proven. A popular theory is that iron is an important regulator of thrombopoiesis. Whereas normal iron levels are required to prevent thrombocytosis by inhibiting thrombopoiesis, a minimum amount of iron is required to maintain platelet production. Thus, thrombocytosis is usually associated with an iron deficiency and is the result of a lack of inhibition of thrombopoiesis. 

**Figure 9:**
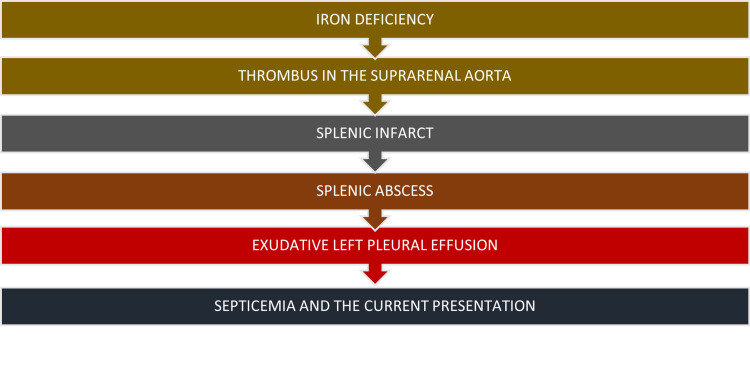
A flow chart of the patient's presentation.

A flow chart simplifying her presentation is given in Figure 9.

A case series report in *The Stroke Journal* by Akins et al. [2] described a scenario where three women with severe iron-deficiency anemia and thrombocytosis secondary to menorrhagia developed carotid artery thrombi. Nagai et al. [3] reported a case of severe iron deficiency with marked thrombocytosis that was complicated by central retinal vein occlusion. Hartfield et al. [6] reported six children with iron deficiency who developed an ischemic stroke or venous thrombosis. Four of them had a concomitant thrombocytosis. The role of iron deficiency anemia in thrombocytosis is hence very much acknowledged but not completely understood. Keung and Owen [4] after undertaking a literature review opined that compared to primary thrombocytosis such as that caused by essential thrombocytosis, reactive thrombocytosis is generally regarded as benign. However, reactive thrombocytosis has been infrequently reported to cause severe and even fatal complications.

## Conclusions

This case is particularly interesting because of the unusual presentation, a multitude of red herrings, and the patient's significant response to treatment with simple iron replacement. As the treating physicians, we are also particularly proud of the fact that we persisted in the search for a diagnosis. If it had not been for this, the patient's underlying Crohn's disease would have been missed and she might have had repercussions later on in life. In the author's opinion, we feel chronic severe iron deficiency anemia with secondary thrombocytosis considered to be a potential prothrombotic state. The significant improvement of the patient with simple replacement of her iron stores shows us that iron replacement should not be delayed in such cases. Lastly, a cause for the initial anemia should always be looked for and persistently investigated. After all, the villain might be hiding in plain sight. 
